# Higher heat shock factor 1 expression in tumor stroma predicts poor prognosis in esophageal squamous cell carcinoma patients

**DOI:** 10.1186/s12967-015-0703-x

**Published:** 2015-10-28

**Authors:** Yuehua Liao, Ying Xue, Lin Zhang, Xinwei Feng, Wanli Liu, Ge Zhang

**Affiliations:** Department of Microbial and Biochemical Pharmacy, School of Pharmaceutical Sciences, Sun Yat-Sen University, No. 132 Waihuandong Road, University Town, Guangzhou, 510006 China; Department of Clinical Laboratory Medicine, Sun Yat-Sen University Cancer Center, Guangzhou, China

**Keywords:** Esophageal squamous cell carcinoma, Heat shock factor 1, Tumor microenvironments, Clinical prognosis, Stromal cell

## Abstract

**Background:**

Heat shock factor 1 (HSF1) is a powerful, multifaceted modifier of carcinogenesis. However, the clinical significance and biologic function of HSF1 in esophageal squamous cell carcinoma (ESCC) remain unknown.

**Methods:**

HSF1 was detected in ESCC cell lines, fibroblast cell lines and ESCC xenograft tumors and human ESCC tissues by real-time RT-PCR and western blotting. HSF1 protein expression was analyzed by immunochemistry in 134 ESCC patients followed by correlation with clinicopathological parameters.

**Results:**

HSF1 expression is weak in fibroblast cell 3T3 and moderate in ESCC cell Eca109, but increasing expression of HSF1 was observed in both of 3T3 and Eca109 cells when they interplayed with each other. In Eca109 xenograft tumors, both tumor cells and stromal fibroblasts showed stronger expression of HSF1. In ESCC patients, the HSF1 expression in tumor or in stromal cells was significantly associated with tumor stage, lymph node metastasis and clinical stage. Multivariate analysis demonstrated a significant negative correlation between disease-free survival (DFS), overall survival (OS) and the HSF1 expression in stromal cells (*P* < 0.05) but not in tumor cells. Additionally, the expression of HSF1 in tumor cells or stromal cells was an independent factor for DFS (*P* = 0.032 or *P* = 0.012) and OS (*P* = 0.017 or *P* = 0.013) in metastatic ESCC patients but not for locoregional ESCC. ESCC patients with low HSF1 in both tumor cells and stromal cells had the longest survivals (*P* < 0.001).

**Conclusions:**

The interaction of tumor cells and stromal fibroblasts increases the expression of HSF1 reciprocally in tumor microenvironment. The HSF1 expression in stromal cells was significantly associated with poor prognosis of ESCC.

## Background

Esophageal squamous cell carcinoma (ESCC), the major histological type of esophageal cancer, is the fourth most frequent cause of cancer deaths in China [1, 2]. Despite general advances in diagnosis and treatment in recent years, ESCC is still disturbing because of the poor prognosis [3]. High rates of recurrence and metastasis facilitate a high mortality rate in ESCC patients. Therefore, there is an urgent need to explore valuable prognostic biomarkers for ESCC patients.

Heat shock transcription factor 1 (HSF1) is a major transcriptional regulator of the heat-shock response (HSR) in eukaryotic cells [4]. HSF1 is evoked in response to a variety of cellular stressors by the upregulation of Hsp70 protein expression. To protect the proteome under diverse physiological stresses, HSF1 governs the cellular response to disruptions in protein homeostasis by influencing fundamental cellular processes, such as cell-cycle control, protein translation, and glucose metabolism [5, 6]. A number of studies have indicated that HSF1 plays a critical role in carcinogenesis, tumor progression and metastasis by regulating the expression of heat shock proteins and other molecular targets [7]. Recently, studies have revealed that HSF1 not only drives transcription in cancer cells but is also capable of reprogramming transcription extensively in cancer-associated fibroblasts (CAFs). As a result, the activation of HSF1 in CAFs promotes malignancy in adjacent cancer cells [8].

Overexpression of HSF1 was observed in a broad range of cancer cell lines and human tumors including colorectal cancer, breast cancer, lung cancer [9], hepatocellular carcinoma [10], endometrial carcinoma [11], oral squamous cell carcinoma [12], glioma [13], melanoma [14] and multiple myeloma [15]. High levels of HSF1 expression in tumor tissues has been reported to be associated with poor progression in patients with breast cancer [16], endometrial carcinoma [11], hepatocellular carcinoma [17] and Hodgkin’s lymphoma [18]. In addition, a study has demonstrated that high expression of HSF1 in peritumoral tissue but not in hepatocellular carcinoma tissue was associated with poorer survival and shorter time to recurrence [17]. Moreover, Scherz-Shouval's study observed increased HSF1 expression in stromal cells but not in tumor cells, which is an indispensable prognostic marker for breast cancer and lung cancer [8]. However, the expression of HSF1 in ESCC and its role in ESCC remain unclear.

In the present study, we measured the expression pattern of HSF1 in different cell populations, including tumor cells and stromal cells, in the tumor microenvironment of ESCC, and investigated their associations with patients’ clinical outcomes, to assess whether HSF1 is a valuable prognostic biomarker for ESCC.

## Methods

### Cell lines

The ESCC cell lines Eca109, Kyse530, Kyse510 and mouse embryo fibroblast cell line NIH 3T3 (Chinese Academy of Sciences, Shanghai, China) were grown in RPMI 1640 (Invitrogen, USA) supplemented with 10 % fetal bovine serum.

### Patients and tissue samples

Eight pairs of ESCC tissue specimens and corresponding non-tumorous specimens were obtained from patients with ESCC who underwent surgical esophageal tissue resection at the Cancer Center of Sun Yat-sen University (Guangzhou, People’s Republic of China) during 2012. All excised samples were obtained within 1 h after the operation from tumor tissue and corresponding non-tumorous tissue 5–10 cm away from the tumor and were immediately kept in liquid nitrogen until further analysis.

In addition, paraffin-embedded tumor tissue samples were obtained from 134 ESCC patients who underwent surgery at Sun Yat-Sen University Cancer Center from May of 2000 to December of 2002. None of the patients had received anticancer treatment prior to surgery, and all of the patients had histologically confirmed primary ESCC in this retrospective study. Clinical information from 134 ESCC samples was described in detail as shown in Table 1. The patients had a median age of 61.5 years (range 33–90 years); 108 (80.6 %) were males and 26 (19.4 %) were females. There were 72 (53.7 %) cases of Stage I and II tumors and 62 (46.3 %) cases of Stage III and IV tumors based on the International Union against Cancer 2002 TNM staging system and WHO classification criteria [19].

**Table 1:** Clinical characteristic of 134 patients with ESCC

Characteristics	No. (%)
Total case	134
Age (years)	
Median	61.15
Range	33–90
Gender	
Male	108 (80.6)
Female	26 (19.4)
Degree of differentiation	
G1	42 (31.3)
G2	57 (42.5)
G3	35 (26.1)
Tumor (T) status	
T1	9 (6.7)
T2	39 (29.1)
T3	81 (60.4)
T4	5 (3.7)
Lymphoid nodal (N) status	
N0	65 (48.5)
N1	69 (51.5)
Distant metastasis (M) status	
M0	128 (95.5)
M1	6 (4.5)
TNM stage	
I	7 (5.2)
IIa–IIb	65 (48.5)
III	56 (41.8)
IV	6 (4.5)
Death	
No	40 (29.9)
Yes	94 (70.1)

The follow-up data from the 134 patients in this study were available and complete. A total of 94 (70.1 %) patients died during the follow-up period. The overall survival (OS) was defined as the time interval from the date of surgery to the date of cancer-related death or the end of follow-up (February, 2012), and the disease-free survival (DFS) was defined as the time interval from the date of surgery to the date of tumor recurrence or tumor metastasis. The diagnostic examinations consisted of esophagography, CT, chest X-ray, abdominal ultrasonography and bone scan when necessary to detect recurrence and/or metastasis.

Prior to the use of all of the clinical materials for investigation, informed consent from patients and approval from the Research Ethics Committee of the Sun Yat-Sen University Cancer Center were obtained.

### Xenograft tumor

The six- to eight-week-old BALB/c-nude mice were provided by Guangdong Medical Laboratory Animal Centre (Guangdong, China) and housed under specific pathogen-free conditions in the Laboratory Animal Center of Sun Yat-sen University. This study was approved by the ethics committee of Sun Yat-Sen University. The mice were inoculated subcutaneously under the right shoulder with 2 × 10^6^ Eca109 cells. After growth for 5 or 7 weeks, the animals were sacrificed, and the xenograft tumors were removed for use.

### Real-time RT-PCR

Total RNA was extracted from cell lines and ESCC tissues were frozen using the Trizol reagent (Invitrogen, USA) according to the manufacturer’s instruction. Reverse transcription of total RNA (2 μg) was performed using SuperScript II reverse transcriptase. The quantification of target and reference glyceraldehyde-3-phosphate dehydrogenase (GAPDH) genes was performed in triplicate on a LightCycler^®^ 480 II (Roche, Applied Science) using a SYBR green-based assay (BioRad, USA). The primers used in the real-time RT-PCR reaction were as follows: HSF-1 forward 5′-ACCCATGCTTCCTGCGTGGC-3′ and reverse 5′- TGCTTCTGCCGAAGGCTGGC-3′; and GAPDH, forward 5′-GACTCATGACCACAGTCCATGC-3′ and reverse 5′-AGAGGCAGGGATGATGTTCTG-3′.

### Western blot analysis

Total protein was extracted using a lysis buffer and protease inhibitor (Beyotime Biotechnology, China). Equivalent protein amounts were denatured in an SDS sample buffer, and then were separated by SDS-PAGE and transferred onto polyvinylidene difluoride membrane. After being blocked with 5 % non-fat dry milk in PBS containing 0.05 % Tween-20, the blotted membranes were incubated with anti-human HSF1 antibody (1:300, Boster, China) and secondary antibody (1:5000, Boster, China) thereafter. GAPDH protein levels were also determined by using the specific antibody (1:1000, Boster, China) as a loading control.

### Immunohistochemistry

The paraffin-embedded tissues were sectioned into 4-μm-thick sections. The sections were dewaxed, rehydrated and rinsed. The antigens were retrieved by heating the tissue sections at 100 °C for 20 min in citrate (10 mmol/L, pH 6.0) solution when necessary. The sections were subsequently immersed in a 3 % hydrogen peroxide solution for 10 min to block endogenous peroxidase activity and were incubated with the primary antibody rabbit anti-human HSF1 (1:40, Boster, China) at 4 °C overnight. A negative control was performed by replacing the primary antibody with PBS. The sections were then incubated with a horseradish peroxidase labeled secondary antibody (1:100, Boster, China) at room temperature for 120 min. Finally, the signal was developed for visualization with 3, 3′-diaminobenzidine tetrahydrochloride, and all of the slides were counterstained with hematoxylin.

### Evaluation of immunohistochemical staining

Two independent observers (Yue-Hua Liao and Xin-Wei Feng) blinded to the clinicopathological information scored the HSF1 expression level in tumor cells and stromal cells by assessing (a) the proportion of positively stained cells (0, <5 %; 1, 6–25 %; 2, 26–50 %; 3, 51–75 %; 4, >75 %) and (b) the intensity of staining (0, negative staining; 1, only cytoplasm staining; 2, low nucleus staining; 3, strong nucleus staining). The score was the product of a × b. The patients were divided into subgroups: a high-level group (a × b ≥ 7 score in tumor cells; or a × b ≥ 6.5 score in stromal cells) and a low-level group (a × b < 7 score in tumor cells; or a × b < 6.5 score in stromal cells) based on the medians of immunohistochemical variable values in diverse cell subsets in our data.

### Statistical analysis

All analyses were conducted with SPSS 16.0 (SPSS Inc., Chicago, IL, USA). Pearson’s Chi-square test and Fisher’s Chi-square test were used to analyze the correlation between HSF1 expression in different cell subsets and the patients’ clinicopathological parameters. HSF1 expression level was examined in tumor cells and in stromal cells in relation to the patients’ clinical prognosis using the Kaplan–Meier method and the log-rank survival analysis. Prognostic factors were assessed by univariate and multivariate analyses using the Cox proportional hazards model. The correlations among the expression levels of HSF1 in tumor cells and in stromal cells were determined using Pearson’s correlation coefficient and linear regression analyses. A two-tailed *P* value <0.05 was considered to be statistically significant in this study.

## Results

### HSF1 expression in ESCC and fibroblast cell lines

**Fig. 1:**
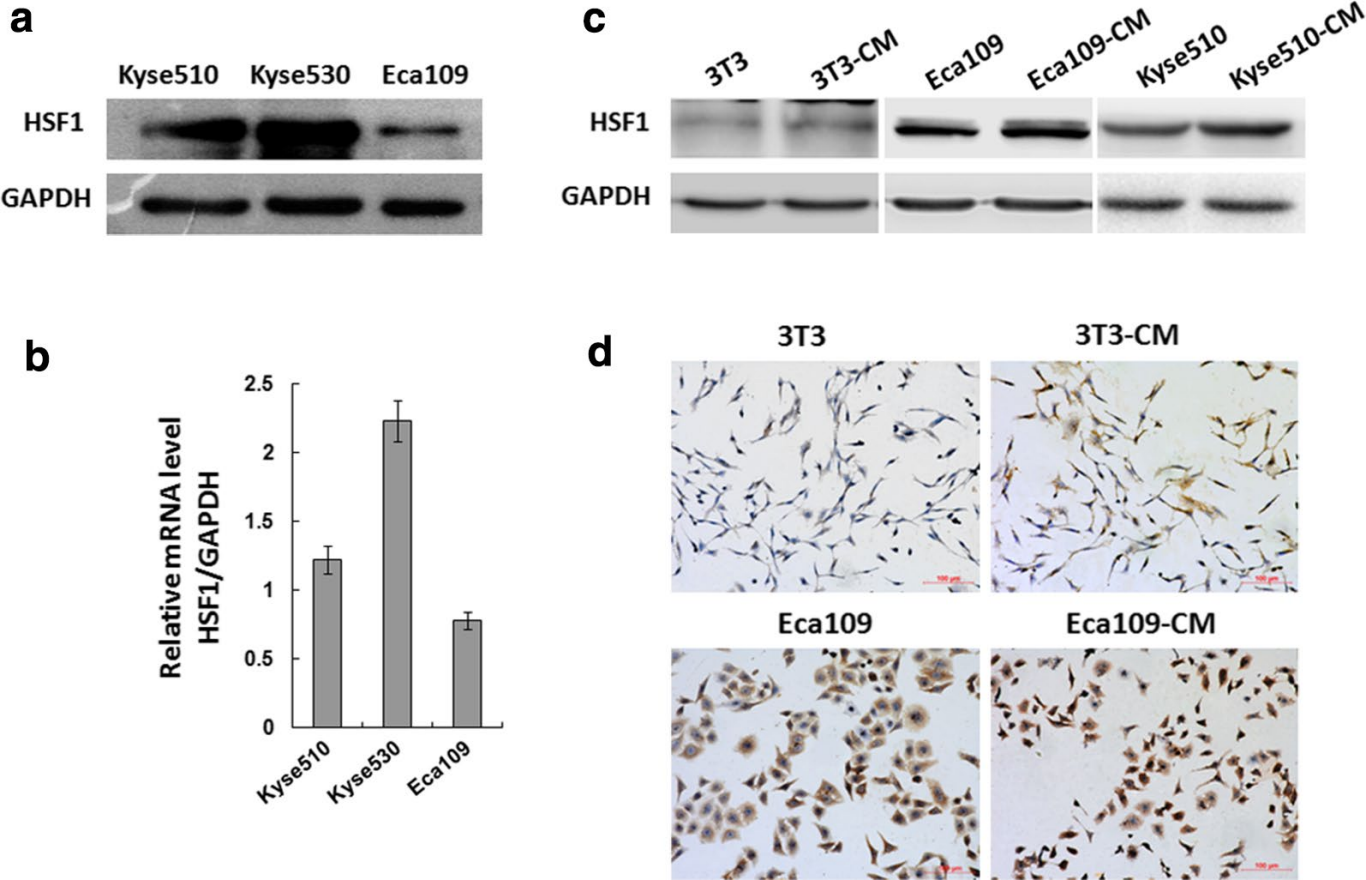
Expression of HSF1 in esophageal squamous cells and fibroblasts. **a** HSF1 protein expression was detected by western blot and **b** HSF1 mRNA expression was detected by qPCR in the ESCC cell lines Eca109, Kyse 510, Kyse 530. **c** HSF1 expression of Eca109 cultured with 3T3-conditioned culture, 3T3 cultured with Eca109-conditioned culture and Kyse510 cultured with 3T3-conditioned culture were detected by western blot. **d** HSF1 expression of Eca109 cultured with 3T3-conditioned culture and 3T3 cultured with Eca109-conditioned culture were detected by immunohistochemical staining (×200)

Western blotting and real-time PCR analysis showed that both HSF1 mRNA and protein were expressed differently in three ESCC cell lines: moderately in Eca109 and strongly in Kyse 510 and Kyse 530 (Fig. 1a, b). Figure 1c showed that HSF1 was less expressed in mice fibroblast cell lines 3T3 by western blotting analysis, compared with the HSF1 expression of Eca109 cell lines and Kyse510 cell lines. Eca109 and 3T3 cells were cultured with conditioned medium of each other reciprocally, and the two cells cultured under hypoxia stress were used as positive control. The same way was used between Kyse510 cells and 3T3 cells. As shown in Fig. 1c, increasing expression of HSF1 was detected in all of these three cells by western blotting. In Fig. 1d, the immunohistochemical results showed the difference between the Eca109 cells and 3T3 cells when they were cultured with conditioned medium or not.

### HSF1 expression in human ESCC xenograft

**Fig. 2:**
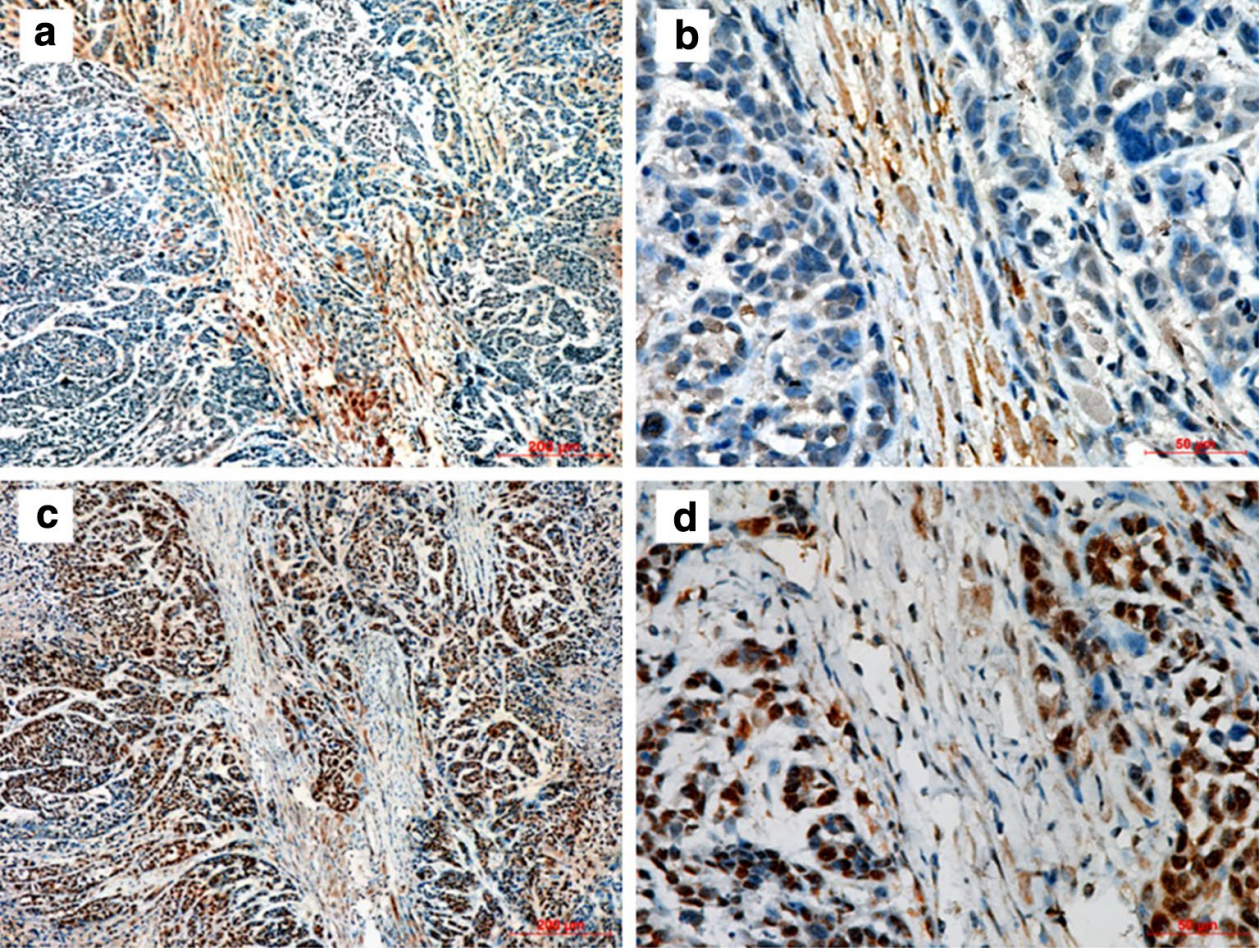
Expression of HSF1 in human Eca109 xenograft tumors. The FAPα staining (**a** ×100; **b** ×400) and HSF1 staining (**c** ×100; **d** ×400) in human Eca109 xenograft tumors

Then, Eca109 cells were inoculated in nude mice to investigate the exact interaction state of HSF1 in vivo. As shown in Fig. 2, the human Eca109 recruited the mice stromal cells into xenograft tumor masses. Eca109 cancer cell nests were surrounded by activated mice fibroblast cells, which were fibroblast activation protein-α (FAPα)-positive by immunohistochemistry (Fig. 2a, b). HSF1 was present in the nucleus mainly in Eca109 tumor cells, and present in the nucleus or distributed between the cytoplasm and a diffuse nuclear localization in stromal fibroblasts (Fig. 2c, d). Both Eca109 tumor cells and the mice stromal fibroblasts showed strong HSF1 positivity in in vivo tumor xenografts. These results indicated that these two cell lines, fibroblast cells and esophageal carcinoma cells, interplay with each other in the tumor microenvironment, which leads to the increasing expression of HSF1 reciprocally.

### HSF1 expression in human normal and ESCC tissues

**Fig. 3:**
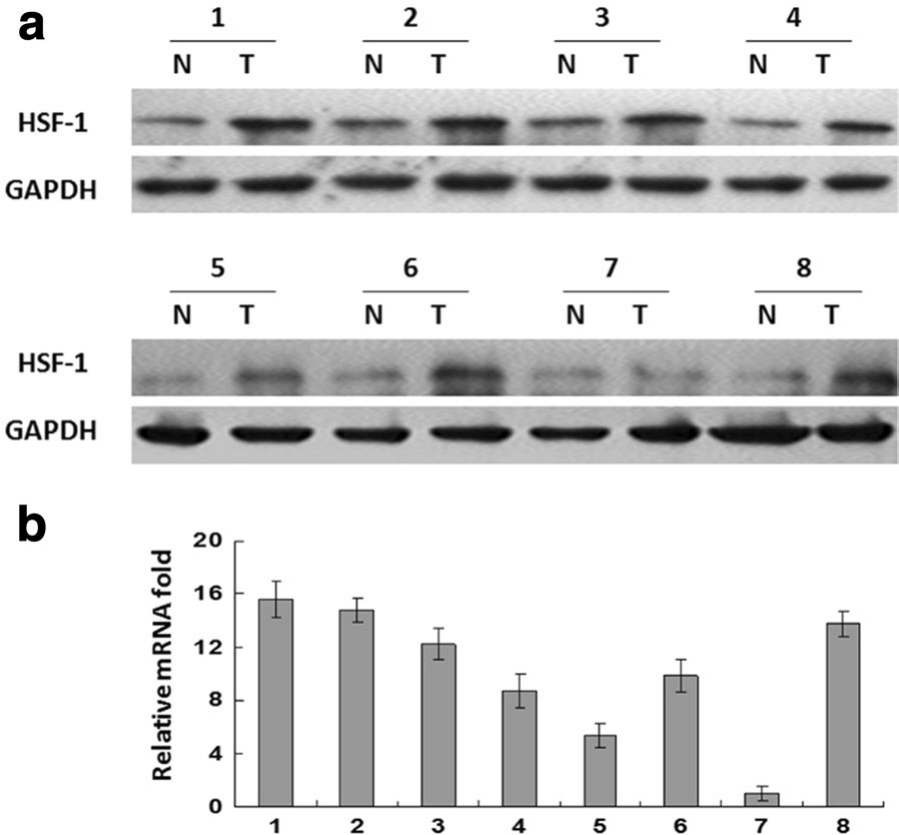
Expression of HSF1 in ESCC carcinoma tissue and matched normal adjacent tissue. **a** HSF1 protein expression was detected by western blot and **b** HSF1 mRNA expression was detected by qPCR in ESCC tumors and normal tissues from four 8 ESCC patients

To investigate the expression of HSF1 in human ESCC tissues, we examined the expression of HSF1 in tumor tissues and matched normal adjacent tissues from eight ESCC patients by western blotting and real-time PCR analysis. As shown in Fig. 3a, in seven of eight cases, more HSF1 was present in the tumors than in the matched controls’ adjacent tissue. The expression of HSF1 in tumor was the same as the expression in normal tissue in only one case. Consistent with the upregulated protein level, HSF1 mRNA expression was also upregulated in tumor tissue compared with the paired non-tumor tissue as analyzed by real-time PCR (Fig. 3b). The tumor/normal (T/N) ratio of HSF1 message signals varied from approximately 1.0- to 15.6-fold in eight paired tissues.

### Expression of HSF1 in ESCC and its correlations with clinicopathological parameters

**Fig. 4:**
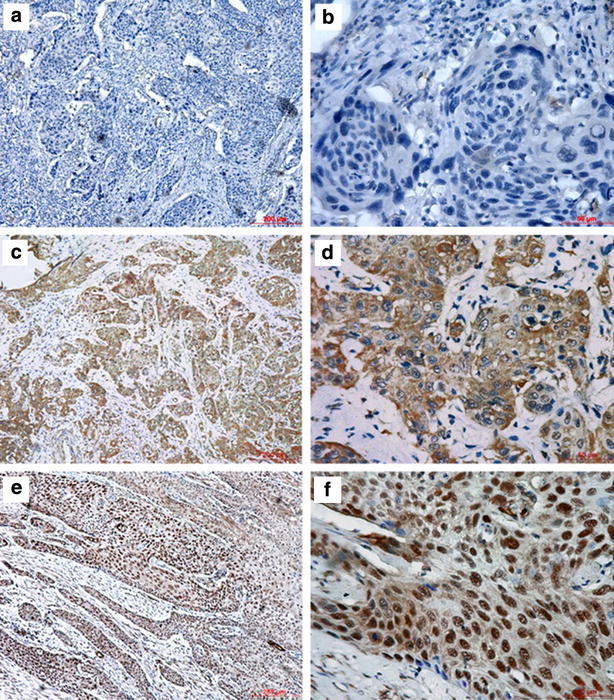
Immunohistochemical staining for HSF1 in tumor cells of ESCC. The negative expression level (**a** ×100; **b** ×400), low expression level (**c** ×100; **d** ×400) and high expression level (**e** ×100; **f** ×400) of HSF1 in tumor tissues from patients with ESCC

**Fig. 5:**
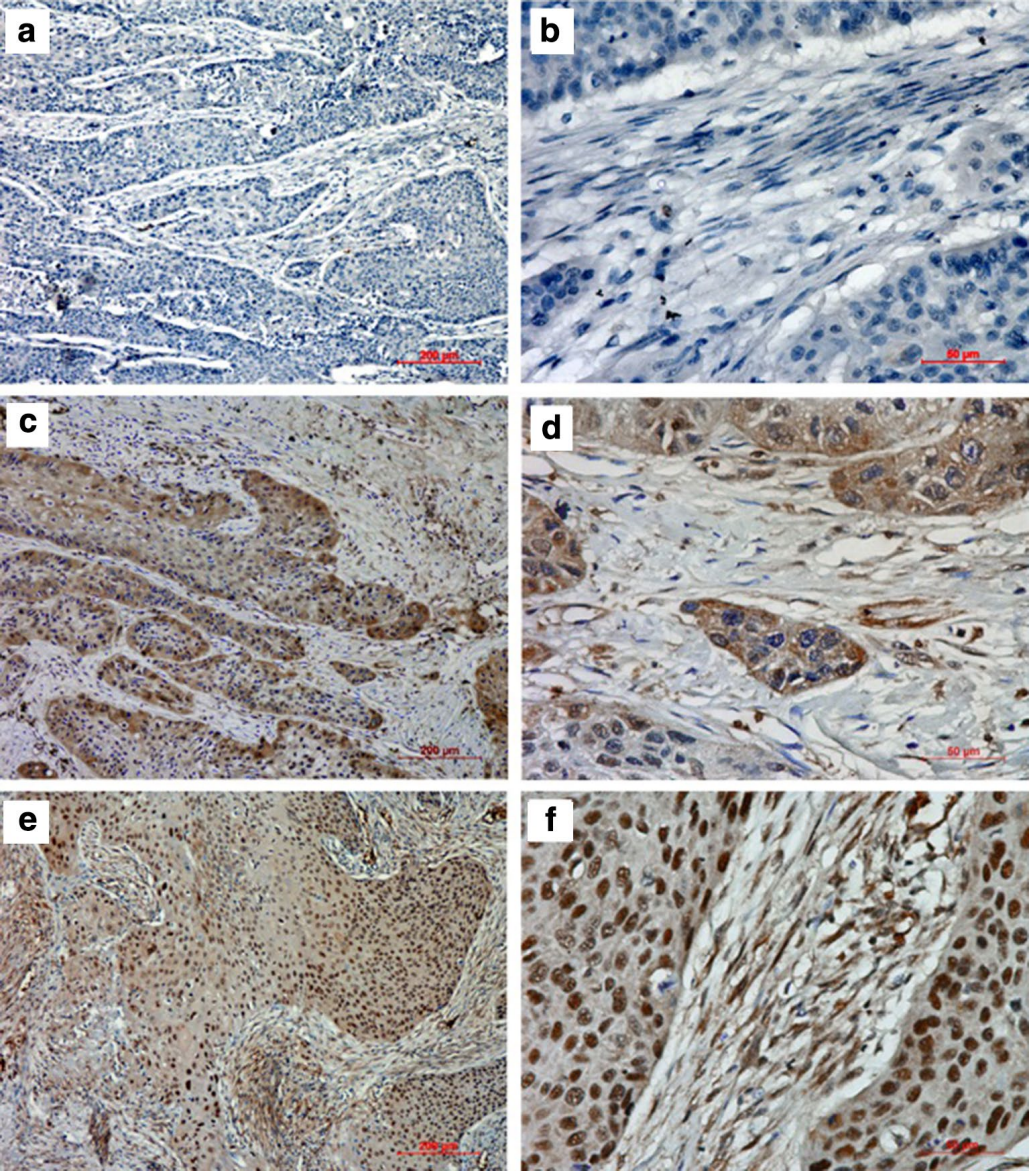
Immunohistochemical staining for HSF1 in stromal cells of ESCC. The negative expression level (**a** ×100; **b** ×400), low expression level (**c** ×100; **d** ×400) and high expression level (**e** ×100; **f** ×400) of HSF1 in tumor stroma from patients with ESCC

To investigate the correlations of HSF1 expression and clinicopathological parameters, the level of HSF1 protein was next determined by immunohistochemistry in 134 archival ESCC tissues. HSF1 protein was detected in 124 of 134 ESCC cases (92.54 %), including 128 cases (95.52 %) in cancer cell nests and 124 cases (92.54 %) in cancer stroma. HSF1 immunoreactivity was observed at various levels, and localization was observed in the nucleus and cytoplasm of both tumor cells (Fig. 4) and stromal cells (Fig. 5). Based on the criteria described in the methods section, a high expression level of HSF1 in tumor cells was noted in samples from 74 (55.2 %) of the 134 patients. As in tumor cells, a high expression level of HSF1 in stromal cells was also found in samples from 74 (55.2 %) of the 134 patients.

The associations between clinicopathological features and HSF1 expression in tumor cells and stromal cells in samples from 134 ESCC patients were summarized in Table 2. High expression level of HSF1 in tumor cells were closely associated with advanced clinicopathological characteristics, including tumor (T) status (*P* < 0.001), node (N) status (*P* = 0.017) and clinical stage (*P* = 0.007), but not significantly associated with age, gender, degree of differentiation and distant metastasis (M) status. Furthermore, the expression level of HSF1 in stromal cells was not only related to the clinicopathological parameters, including T status (*P* < 0.001), N status (*P* = 0.002) and clinical stage (*P* = 0.007), but also related to the gender (*P* = 0.019) and the degree of differentiation (*P* = 0.035). There was no significant correlation between the expression level of HSF-1 protein in stromal cells with age and M status.

**Table 2:** Clinicpathological associations of HSF-1 expression levels in 134 patients with ESCC

Clinicopathologic parameter	Total case (n = 134)	High level of HSF-1 in tumor (%)	P	High level of HSF-1 in stromal (%)	P
Age (years)					
≤61	67	37 (55.2)	0.727	36 (53.7)	0.486
>61	67	39 (58.2)		40 (59.7)	
Gender					
Female	26	11 (42.3)	0.099	10 (38.5)	0.036*
Male	108	65 (60.2)		66 (61.1)	
Degree of differentiation					
G1	42	18 (42.9)	0.082	17 (40.5)	0.021*
G2	57	37 (64.9)		39 (68.4)	
G3	35	21 (60.0)		20 (57.1)	
T status					
T1–2	48	15 (31.2)	<0.001*	15 (31.2)	<0.001*
T3–4	86	61 (70.9)		61 (70.9)	
N status					
N0	65	29 (44.6)	0.006*	28 (43.1)	0.002*
N1	69	47 (68.1)		48 (69.6)	
M status					
M0	128	73 (57.0)	1.000	72 (56.2)	0.935
M1	6	3 (50.0)		4 (66.7)	
Clinical stage					
I–II	72	32 (44.4)	0.002*	33 (45.8)	0.006*
III–IV	62	44 (71.0)		43 (69.4)	

* P < 0.05, as determined by Pearson’s *Χ* ^2^ test

### Expression level of HSF1 in tumor cells and stromal cells and ESCC patient survival

**Fig. 6:**
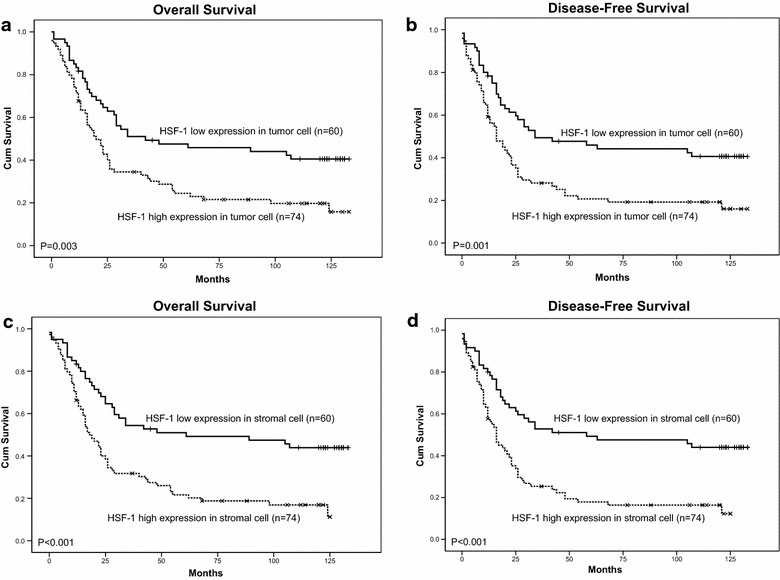
Kaplan-Meier survival analysis in patients with ESCC. **a**, **b** Overall survival and disease-free survival curves for patients according to the low and high expression levels of HSF1 in tumor cells. **c**, **d** Overall survival and disease-free survival *curves* for patients according to the low and high expression level of HSF1 in stromal cells

Among the 134 patients with ESCC, the median survival time was 25 months (range 0–133 months). The cumulative 5-year OS rate and DFS rate of the patients in this study were 32.8 and 28.8 %, respectively. Furthermore, the cumulative ten-year OS rate and DFS rate of the patients in this study were 22.3 and 22.0 %, respectively. Figure 6a,b shows a significant negative correlation between the HSF1 expression in tumor cells and DFS (*P* = 0.001) and OS (*P* = 0.003). Likewise, there was a significant negative correlation between the HSF1 expression in stromal cells and DFS (*P* < 0.001) and OS (*P* < 0.001) (Fig. 6c, d).

The multivariate analysis demonstrated that except for certain conventional clinicopathological parameters, such as gender and nodal status, the HSF1 in stromal cells but not in tumor cells was an independent unfavorable factor for DFS (*P* = 0.019) and OS (*P* = 0.017) (Table 3).

**Table 3:** Multivariate cox regression analysis for OS of 134 patients with ESCC

Variables	OS		DFS	
	HR (95 % CI)	P	HR (95 % CI)	P
HSF-1 expression in tumor cells (n = 134)				
Gender (male/female)	0.352 (0.177–0.700)	0.003*	0.401 (0.203–0.792)	0.009*
Age (≤61/>61)	0.838 (0.546–1.286)	0.419	0.956 (0.607–1.506)	0.846
Degree of differentiation (1/2/3)	1.195 (0.902–1.585)	0.215	1.199 (0.904–1.592)	0.208
Tumor (T) status (1–2/3–4)	0.879 (0.449–1.720)	0.706	0.880 (0.447–1.732)	0.712
Nodal (N) status (0/1)	2.977 (1.464–6.055)	0.003*	2.823 (1.376–5.791)	0.005*
Metastasis (M) status (0/1)	1.350 (0.448–4.066)	0.594	1.392 (0.464–4.179)	0.555
Clinical status (I II/III IV)	0.805 (0.520–1.247)	0.330	0.845 (0.541–1.322)	0.461
HSF1 in tumor cell (low/high)	1.465 (0.916–2.341)	0.111	1.491 (0.928–2.396)	0.099
HSF1 expression in stromal cells (n = 134)				
Gender (male/female)	0.351 (0.177–0.696)	0.003*	0.411 (0.208–0.810)	0.010*
Age (≤61/>61)	0.788 (0.511–1.215)	0.281	0.887 (0.562–1.399)	0.605
Degree of differentiation (1/2/3)	1.143 (0.862–1.514)	0.354	1.157 (0.871–1.536)	0.314
Tumor (T) status (1–2/3–4)	0.724 (0.363–1.441)	0.358	0.736 (0.368–1.472)	0.386
Nodal (N) status (0/1)	2.490 (1.201–5.162)	0.014*	2.330 (1.104–4.918)	0.026*
Metastasis (M) status (0/1)	1.032 (0.334–3.190)	0.956	1.108 (0.364–3.372)	0.856
Clinical status (I II/III IV)	0.904 (0.577–1.414)	0.658	0.959 (0.604–1.523)	0.859
HSF1 in stromal cell (low/high)	2.039 (1.243–3.345)	0.005*	2.000 (1.208–3.311)	0.007*
HSF1 expression in N0 (n = 65)				
Gender (male/female)	0.151 (0.039–0.586)	0.006*	0.199 (0.058–0.680)	0.010*
Degree of differentiation (1/2/3)	1.666 (0.993–2.797)	0.053	1.691 (1.028–2.782)	0.039*
HSF1 in tumor cell (low/high)	0.493 (0.126–1.931)	0.310	0.452 (0.110–1.867)	0.273
HSF1 in stromal cell (low/high)	2.935 (0.634–13.591)	0.168	3.115 (0.712–13.624)	0.131
HSF1 expression in N1 (n = 69)				
HSF1 in tumor cell (low/high)	0.657 (0.235–1.833)	0.422	0.809 (0.292–2.245)	0.684
HSF1 in stromal cell (low/high)	3.038 (1.055–8.753)	0.040*	2.617 (0.893–7.665)	0.079

* P < 0.05

**Fig. 7:**
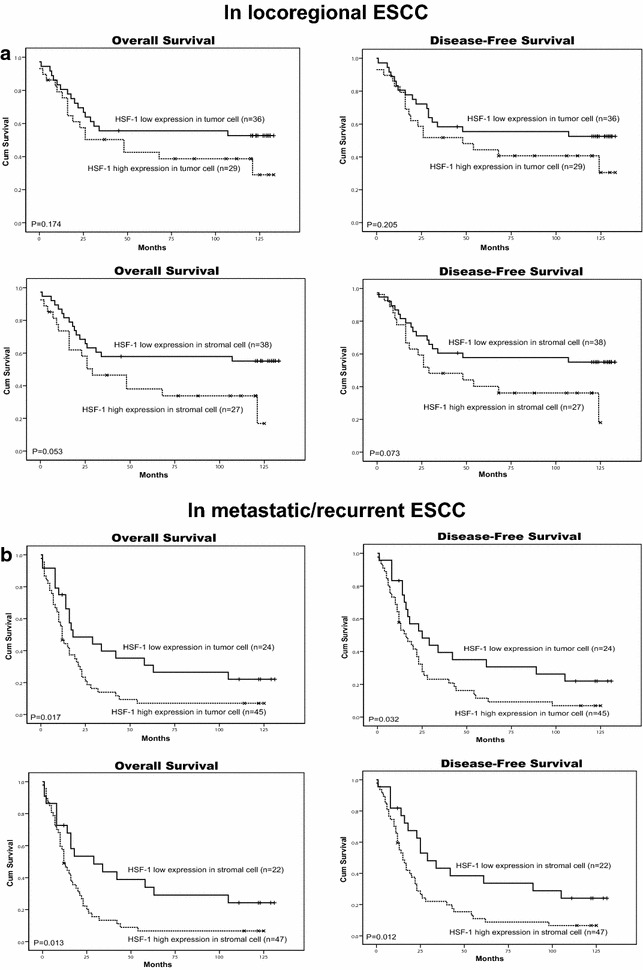
Kaplan-Meier survival analysis in patients with locoregional ESCC and metastatic ESCC. **a** Overall survival and disease-free survival *curves* for patients with low and high expression levels of HSF1 in locoregional ESCC. **b** Overall survival and disease-free survival *curves* for patients with low and high expression levels of HSF1 in metastatic ESCC

Among the 134 patients with ESCC, there were 65 (48.5 %) patients with locoregional ESCC and 69 cases (51.5 %) with metastatic ESCC. The Kaplan–Meier survival analysis showed that the high expression of HSF1 in tumor cells or the high expression of HSF1 in stromal cells was significantly correlated with poor OS (*P* = 0.017, *P* = 0.013) and DFS (*P* = 0.032, *P* = 0.012) in patients with metastatic ESCC (Fig. 7b) but not correlated with poor OS and DFS in patients with locoregional ESCC (Fig. 7a). The results suggested that the expression of HSF1 may be a potential prognostic marker for metastatic ESCC, but not locoregional ESCC.

### The combined expression levels of HSF1 in both tumor cells and stromal cells and the survival of ESCC patients

**Fig. 8:**
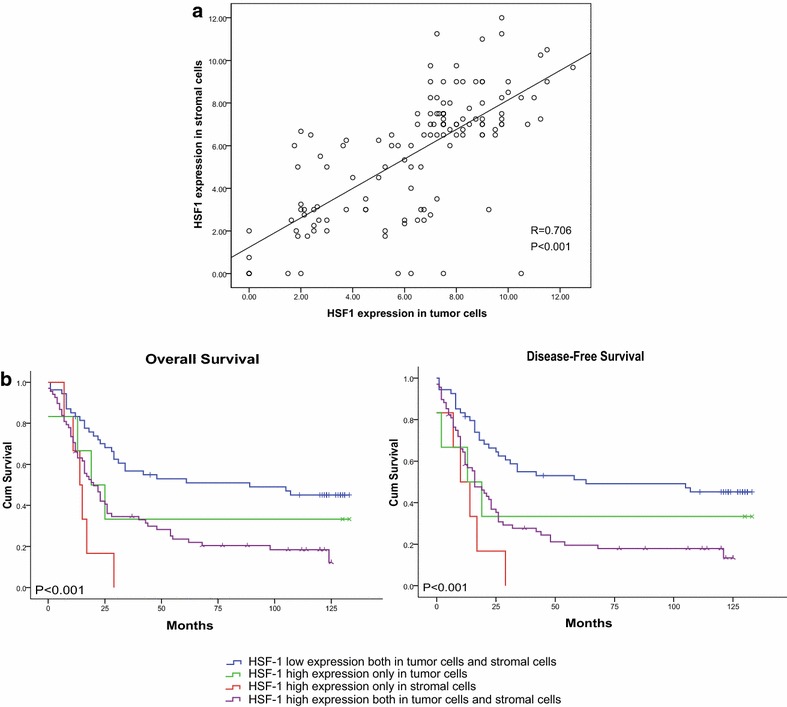
Correlation of HSF1 in diverse cell populations and survival analysis in diverse cell populations. **a** The expression level of HSF1 in tumor cells and stromal cells were significantly positively correlated (*P* < 0.001, R = 0.668). **b** Overall survival and disease-free survival *curves* for patients according to the combined low expression level and combined high expression level of HSF1 in tumor cells and stromal cells, and the single high expression level in tumor cells or in stromal cells

In the current study, Pearson’s correlation coefficient and a linear regression analysis were applied to analyze the correlation between the expression levels of HSF1 in tumor cells and in stromal cells. The HSF1 expression level in tumor cells was positively associated with the HSF1 expression level in stromal cells (*P* < 0.001, R = 0.706; Fig. 8a). Figure 8b, c showed that the patients with a combined low expression level of HSF1 both in tumor cells and stromal cells had the longest DFS and OS, related to those with a single high expression level only in tumor cells or in stromal cells or with a combined high expression level both in tumor cells and in stromal cells. Additionally, the patients with a single high expression level of HSF1 only in stromal cells had the shortest DFS and OS, which indicated that the high expression level of HSF1 in stromal cells is more likely to be the marker for prognosis rather than the high expression level of HSF1 in tumor cells.

## Discussion

In this study, we revealed that the expression of HSF1 was increased in both fibroblast cells and ESCC cells by the interaction in the tumor microenvironment. This finding indicated that the tumor cells and fibroblasts can induce each other reciprocally in HSF1 expression to facilitate tumor progression and metastasis.

Furthermore, we revealed that HSF1 is located in the nucleus and cytoplasm of both tumor cells and stromal cells close to malignant cells in the human ESCC tissues. Strong staining of HSF1 in the nucleus was significantly associated with both advance stage ESCC and poor prognosis in ESCC. Our data were in line with other studies in which high levels of HSF1 expression in the nucleus of several types of cancer, such as breast cancer [16], melanoma and hepatocellular carcinoma, [17] was associated with reduced survival.

HSF1 has historically been viewed as a stress-activated transcription factor. Under basal conditions in normal cells, HSF1 resides primarily in the cytoplasm. Upon activation, it accumulates in the nucleus. In tumors, the high level of HSF1 in the nucleus is a master regulator of protein homeostasis and cell survival to cope with a variety of potentially lethal challenges [20]. It has also been determined that the HSF1 program supports the malignancy of cancer in a variety of ways, including direct effects on the cell cycle, DNA repair, anabolic metabolism, and proliferation [21]. The overexpression of HSF1 in the nucleus of tumor cells indirectly promotes tumorigenesis by enabling proliferation, invasion and metastasis [22, 23]. HSF1 may also play these roles in ESCC and thus account for high levels of HSF1 expression in the nucleus of ESCC tumor cells associated with patients’ poor outcomes.

HSF1 expression was not only found in the ESCC tumor cells but also in stromal cells, primarily close to tumor cells in the microenvironment of ESCC tissues. The increased level of HSF1 in the stromal cells was significantly associated with the clinicopathologic parameters. High levels of HSF1 in stromal cells were correlated strongly with shorter survival in patients with ESCC. These results suggest that the high expression of HSF1 in both tumor cells and stromal cells may be associated with the prognosis of ESCC. Recently, Scherz-Shouval et al. described that overexpression of HSF1 in the CAFs promotes malignancy in adjacent cancer cells. HSF1 activation in the stromal cells correlated strongly with poor outcome in both lung and breast cancer [21]. Zhang JB et al. reported that hepatocellular carcinoma patients with high expression of peritumoral HSF1, but not intratumoral HSF1, have a poor survival, even in patients with slight hepatocellular carcinoma or low α-fetoprotein level. Stromal cells within the tumor microenvironment are essential for tumor progression and metastasis [17]. It is well-known that the cells of the tumor microenvironment contribute to the hallmarks of cancer, and their interaction with cancer cells plays an important role in tumor formation and progression [24, 25]. The microenvironment can provide crucial signaling to maintain tissue architecture, such as the HSR [26]. However, the microenvironment can also promote and induce cancer [27]. If the HSR is governed by the tumor, it may be changed to support cancer cell formation and progression. It is known that HSF1 activation is a key factor in the transcriptional reprogramming of the stroma from a tumor-repressive environment to a supportive one by upregulating genes that promote the malignant phenotype and by downregulating genes that might trigger an anticancer immune response. HSF1 might involve in ESCC microenvironment through the same molecular mechanism. Our dates revealed that only the high expression of HSF1 in stromal cells was related to poor prognosis, rather than the expression of HSF1 in nucleus in tumor cells, which demonstrated that the HSF1 activation in stromal cells was a key factor in the malignant elements. Furthermore, multivariate Cox model analysis showed that only HSF1 expression in stromal cells but not in tumor cells was an independent prognostic marker for ESCC.

Additionally, we observed that high expression of HSF1, whether in tumor cells or in stromal cells, was an independent predictor of DFS and OS in patients with metastatic ESCC, but not locoregional ESCC. This result showed that the HSF1 activation is a more predictive prognostic marker for metastatic ESCC, which means that HSF1 may play a more significant role in tumor migration.

Consistent with these results, in early stage non-small-cell lung cancer and liver cancer patients, HSF1 activation in stromal cells has a more important effect on the progression of these patients than its activation in tumor cells. Why does the expression of HSF1 in tumor cells and in stromal cells show different associations with ESCC? It has been determined out that stromal HSF1 activation drives specific beneficial pathways to the malignant elements, facilitating angiogenesis, ECM organization, adhesion, and migration [20]. HSF1 activation in stromal cells plays a more significant role in the tumor progression or migration and thus may be a better predictor for ESCC patients’ prognosis. In this study, the level of HSF1 expression in tumor cells was positively associated with the HSF1 level in stromal cells. ESCC patients with a combined low expression level of HSF1 both in tumor cells and stromal cells had the longest DFS and OS, whereas the patients with a single high expression level of HSF1 in stromal cells had the shortest DFS and OS, suggesting that the levels of HSF1 activation both in tumor cells and stromal cells and, especially, in stromal cells could improve the ability to predict patient outcome. HSF1 drives a transcriptional program in stromal cells that complements but is completely different from the program it drives in adjacent cancer cells. This stromal cell program driven by HSF1 is uniquely structured to support malignancy in a non-cell-autonomous way. The cooperation between HSF1 activation in stromal cells and tumor cells may promote tumor development. Thus, this cooperation may account for the better outcome of ESCC patients with low levels of HSF1 activation both in stromal cells and tumor cells.

## Conclusions

Our data reveal that the increasing expression of HSF1 is found in ESCC tumor cells and stromal cells reciprocally when they interplay with each other in the tumor microenvironment. Furthermore, the high level of HSF1 expression in both tumor cells and stromal cells was significantly associated with worse DFS and OS of ESCC patients. High HSF1 expression in stromal cells was a better predictor for ESCC patients’ prognosis than its expression in tumor cells, especially in patients with metastatic ESCC. Low levels of HSF1 activation both in stromal and tumor cells predict the best outcome for ESCC patients, suggesting that HSF1 activation is a potential biomarker for ESCC patient prognosis. These findings suggest the possibility of treating ESCC cancer by identifying drugs to targeting HSF1 functions both in the malignant cells and the more genetically stable stroma.

## Abbreviations

HSF1: Heat shock factor 1
ESCC: Esophageal squamous cell carcinoma
CAFs: Cancer-associated fibroblasts
OS: Overall survival
DFS: Disease-free survival
GAPDH: Glyceraldehyde-3-phosphate dehydrogenase
PBS: Phosphate buffered saline
HSR: Heat-shock response
